# In this month’s Bulletin

**DOI:** 10.2471/BLT.24.000324

**Published:** 2024-03-01

In the editorial section, Divya Lakhotia et al. (150) call for papers for an upcoming theme issue exploring digital health solutions for improving health care. Hirotsugu Aiga et al. (151) present this year’s Global Symposium on Health Systems Research.

Gary Humphreys (154–156) reports on World Health Organization’s new estimates of non-melanoma skin cancer deaths. Denis Mukwege talks to Gary Humphreys (157–158) about treating survivors of wartime sexual violence and global efforts to end sexual violence in conflict.

## Angola

### Preventing tuberculosis 

Joan Martínez-Campreciós et al. (196–203) compare contact-tracing strategies.

## American Samoa, Brazil, Cameroon, Côte d’Ivoire, Egypt, Ghana, Guinea, India, Indonesia, Kenya, Liberia, Mali, Nigeria, Papua New Guinea, Samoa, Sierra Leone, Sri Lanka, United Republic of Tanzania

### Molecular xenomonitoring for filariasis

Lisa J Reimer and Joseph D Pryce (204–215) review the evidence for mosquito sampling strategies.

## Burkina Faso

### Diagnosing HIV infection in children

Souleymane Tassembédo et al. (187–195) propose tests to mothers.

## Bangladesh, Brazil, China, Egypt, Haiti, India, Malaysia, Nigeria, Pakistan, Philippines, Türkiye

### Greenhouse gas emissions in health-care systems

Iris Martine Blom et al. (159–175) examine mitigation measures.

## Gambia

### Preventing anaemia in breastfed infants

Mamadou Bah et al. (176–186) trial iron supplements.

## Global

### Machine learning in health financing

Inke Mathauer and Maarten Orange (216–224) delineate benefits, risks and regulatory needs.

